# Surgical Management of Endometrial Polyps in Infertile Women: A Comprehensive Review

**DOI:** 10.1155/2015/914390

**Published:** 2015-08-02

**Authors:** Nigel Pereira, Allison C. Petrini, Jovana P. Lekovich, Rony T. Elias, Steven D. Spandorfer

**Affiliations:** ^1^The Ronald O. Perelman and Claudia Cohen Center for Reproductive Medicine, Weill Cornell Medical College, 1305 York Avenue, 6th Floor, New York, NY 10021, USA; ^2^Department of Obstetrics and Gynecology, Weill Cornell Medical College, 1305 York Avenue, 6th Floor, New York, NY 10021, USA

## Abstract

Endometrial polyps are benign localized lesions of the endometrium, which are commonly seen in women of reproductive age. Observational studies have suggested a detrimental effect of endometrial polyps on fertility. The natural course of endometrial polyps remains unclear. Expectant management of small and asymptomatic polyps is reasonable in many cases. However, surgical resection of endometrial polyps is recommended in infertile patients prior to treatment in order to increase natural conception or assisted reproductive pregnancy rates. There is mixed evidence regarding the resection of newly diagnosed endometrial polyps during ovarian stimulation to improve the outcomes of fresh in vitro fertilization cycles. Hysteroscopy polypectomy remains the gold standard for surgical treatment. Evidence regarding the cost and efficacy of different methods for hysteroscopic resection of endometrial polyps in the office and outpatient surgical settings has begun to emerge.

## 1. Introduction

The interaction between an embryo and a receptive endometrium forms a critical part of early implantation subsequently allowing for placentation and continuation of a healthy pregnancy [1, 2]. It is believed that intrauterine abnormalities, such as polyps, leiomyomata, or synechiae, may perturb this important event [2, 3]. Although isolated uterine-associated infertility can be found in 2-3% of infertile women [4], intrauterine lesions may be found in approximately 40–50% of subfertile or infertile women [4–6]. Lesions such as endometrial polyps have previously been implicated in the pathogenesis of subfertility and early pregnancy loss, though this association is sometimes debated [5, 6]. Previous observational studies have suggested that resection of endometrial polyps can help increase natural conception rates as well as increase pregnancy rates with assisted reproduction [4]. In this paper, we review the epidemiology, pathogenesis, diagnosis and management of endometrial polyps in infertile women. We also critically evaluate the current evidence related to resection of endometrial polyps and its impact on natural conception and assisted reproductive pregnancy rates.

## 2. Epidemiology

Endometrial polyps are benign localized outgrowths of the endometrium that contain glands and stroma [5, 7]. They may occur as single or multiple lesions, can be sessile or pedunculated, and may range in size from millimeters to centimeters [2, 7]. Occasionally, endometrial polyps may contain smooth muscle fibers in addition to glands and stroma and are called adenomyomatous polyps [8]. The true incidence of endometrial polyps remains unknown incidence due to its asymptomatic nature [9]. However, depending on the type of population studied, the prevalence of endometrial polyps can vary from 7.8% to 34.9% [10, 11]. Some studies have reported that endometrial polyps can be found in up to 24% of symptomatic women [12, 13]. The prevalence of endometrial polyps is thought to be higher in infertile women [9]. In a prospective study of 1000 patients undergoing hysteroscopic evaluation of the uterine cavity prior to in vitro fertilization (IVF), the prevalence of endometrial polyps was found to be 32% [14]. While this may suggest a causal association between polyps and infertility, this association has been confirmed in only one randomized control trial so far [15].

## 3. Pathogenesis

Endometrial polyps are rarely diagnosed before menarche [16] suggesting that estrogenic stimulation of the endometrium plays a crucial role in the pathogenesis of endometrial polyps [17]. As these polyps contain immature or functional endometrium, they can develop in conditions associated with increased or unopposed estradiol levels, as in the case of ovarian stimulation during IVF [18]. Age, hypertension, obesity, and diabetes are well known risk factors for the development of endometrial polyps [19, 20]. Of these risk factors, age is perhaps the most well-known risk factor [9]. The prevalence of endometrial polyps increases with age, though it is unclear whether this trend continues past menopause [9]. There also appears to be an association between endometrial polyps and other benign gynecologic conditions such as cervical polyps and endometriosis [21, 22]. Women using tamoxifen are also known to have a higher risk of developing endometrial polyps, and the prevalence of polyps in this patient population is estimated to be between 30 and 60% [23]. Molecular mechanisms such as the overexpression of estrogen and progesterone receptors [24], endometrial aromatase [25, 26], increased B-cell lymphoma 2 protein expression [27], and mutations in the* HMGIC* and* HMGI[Y]* genes [28, 29] have also been implicated in the development of endometrial polyps. Atypical hyperplasia and endometrial cancer may arise in up to 6.7% and 2.2% of endometrial polyps, respectively [30–33]. The risk of malignancy increases with age [9], polyp size [34], and concomitant use of tamoxifen [35].

## 4. Diagnosis

Patients with symptomatic endometrial polyps usually present with abnormal uterine bleeding [36]. However, a large majority of polyps are asymptomatic and are incidentally discovered [2, 9]. The diagnostic modalities that are commonly utilized to diagnose endometrial polyps include 2-dimensional transvaginal sonography (2D TVUS), 3-dimensional transvaginal sonography (3D TVUS), saline infusion sonography (SIS), hysterosalpingography (HSG), and hysteroscopy [37].

### 4.1. 2-Dimensional Transvaginal Sonography

An endometrial polyp usually appears as a hyperechoic endometrial mass with regular contours occupying the uterine cavity either partially or fully [15]. Occasionally, cystic spaces may appear within the polyp [38]. Performing sonography in the proliferative phase of the menstrual cycle often provides the most reliable results [39]. 2D TVUS, in experienced hands, can detect endometrial polyps accurately [40]. In a large study of 793 women, the sensitivity, specificity, positive predictive value (PPV), and negative predictive value (NPV) of 2D TVUS in detecting endometrial polyps were found to be 86%, 94%, 91%, and 90%, respectively [41]. The addition of color-flow Doppler can improve the diagnostic capability of 2D TVUS by allowing visualization of the single feeding vessel present in endometrial polyps [9]. Color-flow Doppler, in some studies, has shown to increase the sensitivity of 2D TVUS from 91% to 97% [42].

### 4.2. 3-Dimensional Transvaginal Sonography

Compared to 2D sonography, 3D TVUS with color-flow Doppler allows for the measurement of endometrial volume, as well as endometrial and subendometrial vascularization indices [43, 44]. Some studies have suggested that using a combination of endometrial echogenicity, thickness, and volume with 3D TVUS may be better than single measurements with 2D TVUS for detecting endometrial polyps [43]. In contrast, others have shown that noncontrast 3D TVUS does not necessarily increase detection of endometrial polyps compared to 2D TVUS [45].

### 4.3. Saline Infusion Sonography

The addition of intrauterine contrast (saline or gel) increases the diagnostic accuracy of 2D TVUS and 3D TVUS [9]. Additional advantages of SIS include assessment of other uterine cavity abnormalities such as leiomyomata or adhesions and assessment of Müllerian anomalies, if needed [37, 46, 47]. Disadvantages of SIS are related to its learning curve [48] and patient discomfort caused by fluid instillation or leakage [49], as well as the theoretical risk of infection [50]. In a recent systematic review and meta-analysis of 20 studies comparing the diagnostic accuracy of SIS to hysteroscopy, the pooled sensitivity and specificity of SIS in the detection of all intrauterine abnormalities were 88% (95% confidence interval [CI]: 85%–90%) and 94% (95% CI 93%–96%), respectively [37]. Overall, most studies reveal no significant difference between SIS and diagnostic hysteroscopy in diagnosing endometrial polyps [37]. Comparisons between 2D SIS and 3D SIS have also been made [51, 52]. In one such study [51], the sensitivity, specificity, PPV, and NPV for 2D SIS in detecting intrauterine lesions were found to be 71.2%, 94.1%, 90.2%, and 81.0%, respectively. The overall accuracy was 84.2%. For 3D SIS, the sensitivity was 94.2%, specificity 98.5%, positive predictive value 98.0%, negative predictive value 95.7%, and overall accuracy 96.7%. The investigators concluded that 3D SIS was superior to 2D SIS and was comparable to hysteroscopy in diagnosing intrauterine lesions.

### 4.4. Hysterosalpingography

HSG allows imaging of the cervical canal, uterine cavity, and fallopian tubes with injection of contrast media using fluoroscopic visualization [53]. In general, the cervical canal or endometrial cavity is accessed using aseptic technique [53]. A small volume (10–30 mL) of contrast agent is administered under intermittent fluoroscopy to visualize the structures to be imaged [53]. Occasionally, postdrainage images can be obtained when endometrial pathology is suspected [53]. HSG has high sensitivity (98%) but low specificity (34.6%) and PPV (28.6%) compared with hysteroscopy for endometrial polyps [54, 55].

### 4.5. Hysteroscopy

Hysteroscopy with guided biopsy is considered the gold standard for diagnosing endometrial polyps [9, 56]. Hysteroscopy also facilitates assessment of size, number, and vascular characteristics of endometrial polyps [9]. Prior to the routine use of hysteroscopy, blind dilation and curettage were used for the diagnosis of endometrial polyps [57]. This technique, however, caused polyp fragmentation making histopathologic diagnosis difficult [58]. The low sensitivity of 8% to 46% and NPV of 7% to 58% of blind endometrial sampling compared to hysteroscopy with guided biopsy [59] suggests that the former technique should not be used for diagnosing endometrial polyps.

## 5. Treatment Options

### 5.1. Expectant Management

The natural course of endometrial polyps is not well understood [13, 60]. Given that most polyps are benign, expectant management is a reasonable option in asymptomatic premenopausal women [9]. Small endometrial polyps (<10 mm) are thought to regress spontaneously in about 25% of cases [61, 62].

### 5.2. Medical Management

There is limited role of medical management for endometrial polyps [9, 62]. Levonorgestrel containing intrauterine devices have been used to reduce the incidence of tamoxifen-related endometrial polyps in some research settings [63]. Gonadotropin releasing hormone agonists have also been used as an adjunctive treatment before hysteroscopic resection [64]. However, there is little-to-no data supporting its utility as a first-line agent for treating endometrial polyps [62, 64].

### 5.3. Surgical Management

While endometrial polyps may resolve spontaneously and could possibly be amenable to hormonal therapy, definitive treatment options are largely surgical. Blind dilation and curettage can remove endometrial polyps in up to 8% of patients [9]. Addition of polyp forceps increases complete extraction of polyps in approximately 41% of patients [9]. In general, blind dilation and curettage can miss endometrial pathology in approximately 50% of cases and should therefore be avoided when hysteroscopy is available [57–59].

Hysteroscopic polypectomy remains the gold standard for both the diagnosis and treatment of endometrial polyps [9]. The choice of performing hysteroscopy in the office or outpatient surgical setting is generally dependent on patient preference, physician skill, and instrument availability [9, 65]. While equivalent success rates have been reported in both settings, some data indicate that failure to remove a polyp is more likely in the office setting [65]. In contrast, other data suggest that office-based hysteroscopic polypectomy is safe and feasible in patients with endometrial or isthmic polyps < or = 20 mm, independent of menopausal status or previous vaginal delivery [66].

Several hysteroscopic systems to resect endometrial polyps are currently available, monopolar loop cautery [9], bipolar systems [67], microscissors or graspers [9], and hysteroscopic morcellators [68, 69]. Of these, the monopolar loop is more commonly available and of lower cost [9]. Comparative studies about the aforementioned methods with regard to costs and efficacy have recently begun to emerge. For example, in a prospective, randomized study of 100 patients comparing monopolar to bipolar electrode excision of endometrial polyps, the former technique was found to be better for nonfundal polyps or those >20 mm compared to the latter technique, which was better for small, fundal polyps [70]. In another randomized study of 121 patients, removal of polyps using a hysteroscopic morcellator was found to be significantly quicker, less painful, more acceptable to women, and more likely to completely remove endometrial polyps compared to electrosurgical resection [71]. It is important to note that none of these studies were performed in infertile patients. The overall method of hysteroscopy polypectomy is generally the one which the clinician is trained with and most familiar with [9]. The risk of intrauterine adhesions after hysteroscopy polypectomy is low as the myometrium is generally not incised [72]. Other procedural risks associated with hysteroscopy polypectomy include infection, surgical bleeding, uterine perforation, fluid overload, or anesthesia-related complications.

## 6. Impact on Fertility

The putative mechanisms by which endometrial polyps adversely impact fertility may be related to mechanical interference with sperm transportation or as space occupying lesions interfering with embryo implantation [5]. The glands and stroma in endometrial polyps are unresponsive to progesterone stimulation, leading to defective implantation at the site of the polyp [8]. Endometrial polyps may also induce local inflammatory changes, which can interfere with normal implantation and embryonic development [6, 73]. These inflammatory changes are mediated by increased number of mast cells in the endometrial cavity [74], as well as increased levels of matrix metalloproteinase-2 and metalloproteinase-9 [75]. Endometrial polyps can produce glycodelin, a glycoprotein that has been shown to inhibit natural killer cell activity, rendering the endometrium less receptive to implantation [2, 76]. It is also speculated that endometrial polyps decrease messenger RNA levels of* HOXA10* and* HOXA11*, which are known molecular markers of endometrial receptivity [7].

### 6.1. Natural Conception

Previous observational studies have shown that resection of endometrial polyps can improve natural conception rates, particularly in patients with unexplained infertility [3, 5]. In one retrospective study of 78 patients, a pregnancy rate of 78.3% was noted after polypectomy compared to a pregnancy rate 42.1% in patients with normal uterine cavities [77]. Similarly, natural conception rates of 76% [73] and 50% [78] were reported after resection of endometrial polyps. In subfertile women, hysteroscopic polypectomy can improve fertility, with pregnancy rates ranging from 43% to 80% [73, 79].

### 6.2. Intrauterine Insemination

Studies have shown that pregnancy rates are improved in patients undergoing polypectomy before undergoing intrauterine insemination (IUI) [2]. In one prospective randomized study involving 215 patients, patients who underwent polypectomy prior to IUI had an increased pregnancy rate (51.4%) compared to patients who did not (25.4%) [15]. These findings were similar to another independent study, which reported pregnancy rates of 40.7% and 22.3% in patients who did and did not undergo polypectomy before IUI, respectively [80]. Hysteroscopic resection of endometrial polyps (~16 mm) prior to IUI for unexplained male or female factor has also been shown to increase the odds of clinical pregnancy (odds ratio 4.4, 95% CI 2.5–8.0) for at least 2 years, compared to diagnostic hysteroscopy and polyp biopsy alone [81].

### 6.3. In Vitro Fertilization

Current evidence supports the resection of endometrial polyps diagnosed prior to commencement of IVF cycles [6]. The time interval between hysteroscopic resection of polyps and the subsequent IVF cycle does not seem to impact the success rates of the IVF cycle [82]. However, the management of newly diagnosed endometrial polyps during ovarian stimulation still remains controversial [2, 5]. Some studies have shown that resection of newly diagnosed endometrial polyps during COH can decrease rates of pregnancy loss [18] and increase clinical pregnancy [83, 84] and live birth rates [77], while others have shown no such benefits [85, 86]. In one of the earliest studies, 83 patients with polyps <20 mm diagnosed during ovarian stimulation were divided into two groups [18]. The first group (49 patients) underwent IVF with fresh embryo transfer (ET), while the second group (34 patients) underwent hysteroscopic polypectomy immediately after oocyte retrieval. The cryopreserved embryos were thawed and transferred in a subsequent cycle. No difference in pregnancy rates was noted between the two groups. At least 2 other studies have confirmed the aforementioned findings [85, 86]. These findings suggest that endometrial polyps <20 mm during fresh IVF-ET cycles can be managed expectantly without compromising clinical pregnancy or live birth rates [2]. This was further confirmed by a retrospective study that showed no difference in the implantation, clinical pregnancy, or live birth rates after fresh IVF-ET cycles when patients with newly diagnosed endometrial polyps were compared to those with normal endometrial stripes [87]. Most recently, our group [2] reported that newly diagnosed endometrial polyps (<20 mm) during ovarian stimulation are associated with an increased biochemical pregnancy rate, without adversely impacting clinical pregnancy or live birth rates after fresh IVF cycles. Thus, one may hypothesize that small endometrial polyps can create a hostile environment for early embryo development; however, if the embryo does overcome this initial insult, the risk of future miscarriage is primarily related to embryonic aneuploidy or other endometrial factors [2].

## 7. Conclusions

Endometrial polyps are commonly seen in infertile women [6]. The overall evidence suggests a detrimental effect of polyps on fertility [6]. Conservative management of small and asymptomatic polyps is reasonable in most cases [9]. However, surgical resection of endometrial polyps is recommended in infertile patients to possibly increase natural conception and assisted reproductive pregnancy rates [9]. Hysteroscopic polypectomy remains the gold standard for surgical treatment, though evidence regarding the cost and efficacy of different methods for hysteroscopic resection of polyps in the inpatient and outpatient settings has begun to emerge. Management of newly diagnosed endometrial polyps during IVF should be individualized according to the patient's reproductive history, polyp size and location, ovarian stimulation response, the number of good quality embryos, and the individual clinic's success rate with cryopreserved embryo transfer [2, 6].
